# Toxicity, Anti-Inflammatory, and Antioxidant Activities of Cubiu (*Solanum sessiliflorum*) and Its Interaction with Magnetic Field in the Skin Wound Healing

**DOI:** 10.1155/2022/7562569

**Published:** 2022-03-10

**Authors:** Jéssica Franco Dalenogare, Marina de Souza Vencato, Greice Franciele Feyh dos Santos Montagner, Thiago Duarte, Marta Maria Medeiros Frescura Duarte, Camila Camponogara, Sara Marchesan Oliveira, Marcelo Leite da Veiga, Maria Izabel de Ugalde Marques da Rocha, Maria Amália Pavanato, Liliane de Freitas Bauermann

**Affiliations:** ^1^Department of Physiology and Pharmacology, Federal University of Santa Maria, Santa Maria, Brazil; ^2^Department of Morphology, Federal University of Santa Maria, Santa Maria, Brazil; ^3^Department of Biochemistry, Federal University of Santa Maria, Santa Maria, Brazil

## Abstract

Cubiu, an Amazonian fruit, is widely used as food and popular treatment for pathologies that present an inflammatory pattern, such as skin wound healing. However, there is still no confirmation in the scientific literature about the safety profile, as well as the anti-inflammatory, antioxidant, and healing actions of cubiu. This study is divided into two experimental protocols using Wistar rats. Thus, the first objective (protocol 1) of this study was to evaluate the toxicity of an oral administration of cubiu extract at different doses for 28 days. The macroscopic and microscopic analyses of the liver and kidney were performed, and the following analysis was determined in plasma: glutamic oxaloacetic transaminase, glutamic pyruvic transaminase, gamma-glutamyl transpeptidase, glucose, triglycerides, total cholesterol, urea, creatinine, and uric acid. After, we conducted the second protocol aimed to establish the potential antioxidant and anti-inflammatory capacity of cubiu and its interaction with magnetic field in skin wound healing. On days 3, 7, and 14 of treatment, skin and blood samples were collected and analyzed: the oxidative stress biomarkers (reactive substances to thiobarbituric acid, nonprotein thiols, superoxide dismutase, catalase, and glutathione S-transferase), myeloperoxidase enzymatic activity, and cytokines levels (interleukin 1, interleukin 6, interleukin 10, and tumor necrosis factor-alpha). The cubiu has shown to be safe and nontoxic. Both cubiu and magnetic field promoted decreased levels of proinflammatory and prooxidant biomarkers (interleukin 1, interleukin 6, tumor necrosis factor-alpha, and reactive substances to thiobarbituric acid), as well as increased levels of anti-inflammatory and antioxidant biomarkers (interleukin 10, nonprotein thiols, and superoxide dismutase), with greater potential when treatments are used in association. Thus, cubiu promotes antioxidant and anti-inflammatory action in skin wound healing, while also improving results of the conventional treatment for skin healing (magnetic field) when used in association.

## 1. Introduction

*Solanum sessiliflorum* (Dunal) is a Solanaceae native of the Amazon Basin, currently found throughout the Brazilian, Ecuadorian, Colombian, and Venezuelan Amazonian territory. Its fruit, called cubiu in Portuguese, also known as “apple/peach tomato, is widely used as food, and it can be used in numerous ways such as juices, sweets, jellies, and consumed in natura [1]. Furthermore, cubiu is very nutritious and rich in iron, niacin, citric acid, and pectin. Thus, cubiu presents compounds considered adjuvants for health promotion, such as fibers, minerals, and bioactive compounds like phenolic acids [2].

Amazonian people commonly employ cubiu as a remedy or cosmetic. In their traditional medicine system, cubiu is used because of its hypoglycemic, hypolipidemic, and antioxidant properties to treat diabetes and skin wound healing [3–5]. However, no studies have investigated the anti-inflammatory properties of cubiu, considering the inflammatory profile of the pathologies traditionally treated with this plant. Moreover, the in vivo antioxidant potential of cubiu is scarcely approached in scientific literature. Cubiu also could be a suitable alternative for use in combination with magnetic fields. Therapy with magnetic fields is used for skin wound healing, but its systemic performance in the oxidative and inflammatory profile is controversial in the scientific literature. Considering these data, an investigation of magnetic fields associated with antioxidants was suggested [6, 7]. Among these substances, there are natural antioxidants, primarily contained in plants. As previously mentioned, cubiu is commonly used for its antioxidant properties [8] and also popularly used for skin wound healing. Therefore, we believe to be pertinent to elucidate the effects of cubiu combined with magnetic field therapy.

Thus, the first objective of the present study was to evaluate the oral toxicity of cubiu. After completing this objective, the potential antioxidant and anti-inflammatory capacities of cubiu and its interaction with magnetic fields in an in vivo model of skin healing were analyzed.

## 2. Materials and Methods

### 2.1. Cubiu Extract Preparation

Cubiu samples were purchased in the Municipal Market Adolfo Lisboa-Manaus city, Amazonas, Brazil. Botanic specialist Eduardo Vellez Marin (CRBio 09112-03) confirmed the fruits to be *Solanum sessiliflorum* Dunal. This research is part of a project previously authorized by the Brazil Environmental Ministry to assess the components of genetic patrimony in national territory (no. 010547/2013-4) according to Brazilian legislation (no. 2186-16). The cubiu samples were registered in the Management of Genetic Patrimony Council, Brazil (CGEN, process number A6723EB).

The fresh fruits of cubiu weighed 147 ± 38 g. The preparation of the cubiu extract included washing and peeling the fruits, as well as grinding the pulp with small seeds in a mixer for 5 min and then extracting with 70% absolute ethanol (neon, commercial-03467; São Paulo, SP, Brazil). After extraction, the product obtained was filtered, evaporated, lyophilized, and was stored in a −20 degrees freezer [9]. Subsequently, the lyophilized cubiu extract was diluted daily in saline and administered via oral gavage for oral administration. The use of fresh fruit followed institutional, national, and international guidelines and legislation. The cubiu extract phytochemical characterization is demonstrated in the study by Montagner et al. [9].

### 2.2. Experimental Animals

Male Wistar rats (90 days of life, weighing 150 g) were obtained from the Central Animal Facility of the Federal University of Santa Maria (UFSM) and were maintained during the experimental protocol at the Animal Facility of the Physiology Department (UFSM), under controlled environmental conditions (23°C ± 1), 12-hour light/dark cycle, and food and water provided ad libitum. This research was conducted following the Animal Research: Reporting of In Vivo Experiments (ARRIVE guidelines) and the internationally accepted guidelines on animal welfare (EEC Directive 1986; 86/609/EEC) and in agreement with national and institutional rules. The experimental protocol was approved by the Ethics Committee on Animal Use of the Federal University of Santa Maria, registration no. 119/2013.

### 2.3. Protocol 1: Toxicity Evaluation of Cubiu Extract by Dose-Response Curve

A curve was proposed for different doses of the cubiu extract diluted in saline and administrated by oral gavage for 28 days (*n* = 3 animals per group, total = 12 animals). This allowed verifying the toxicity of the cubiu extract and the most adequate or beneficial dose for protocol 2. Tested doses of cubiu extract were 25 mg/kg, 50 mg/kg, 100 mg/kg, and 150 mg/kg per rat body weight. This protocol had a control group that received only saline. The toxicity was performed according to OECD guideline 407 (OECD, 2008) [10] with slight modifications.

#### 2.3.1. Biochemical Analyses

Plasma analyses of glutamic oxaloacetic transaminase (GOT), glutamic pyruvic transaminase (GPT), gamma-glutamyl transpeptidase (GGT), glucose, triglycerides, total cholesterol, urea, creatinine, and uric acid were performed using a commercial kit (Bioclin®), according to manufacturer's instructions. These analyses were processed in an automatic biochemical analyzer (Mindray BS-120®). The results were expressed in UI/l (GOT, GPT), U/l (GAMA GT), mg/dl (triglycerides, total cholesterol, urea, creatinine, and uric acid), and g/dl (glucose).

#### 2.3.2. Morphological Analyses

Organs were evaluated macroscopically regarding preservation of organ architecture, presence of hemorrhage, and any aspects associated with degeneration, in addition to, shape, size, color, and general appearance. After euthanasia, the samples were fixed in 10% buffered formalin and embedded in histological paraffin for light microscopic examination after removal. Sections (6 *μ*m thick) were stained using the hematoxylin and eosin method. Two independent observers performed double-blind analysis. For liver samples, following criteria were evaluated: presence of inflammatory infiltrate, cellular degeneration or necrosis, congestion or dilatation of the central vein or liver sinusoids, and presence of sinusoidal vacuolization. For kidney samples, evaluated criteria were dilatation of Bowman's capsule, dilatation of subcapsular space, dilatation of proximal and distal convoluted tubules, and glomerular and tubular degeneration [11].

#### 2.3.3. Oxidative Status

Evaluation of oxidative status was conducted in homogenized hepatic and renal tissue. To homogenize tissue samples, 9 mL of sodium phosphate buffer 0.3M (KCl 140 mM, pH 7.4) was used per gram of tissue. Phenylmethylsulfonyl fluoride (PMSF) was added at the concentration of 100 nM diluted with isopropanol, 10 *μ*l of PMSF per mL of buffer. Homogenization was performed for 60 seconds in ULTRA-TURRAX at 0–4°C. Centrifugation of homogenate was carried in refrigerated equipment for 10 minutes at 3000 rpm (1100 g).

*(1) Total Protein Determination*. Protein content in the homogenate was evaluated using the method described by Lowry et al. [12]. Results were expressed in mg/ml.

*(2) Thiobarbituric Acid Reactive Substances (TBARS) Determination*. The TBARS method was carried in accordance with Buege and Aust [13]. Results were expressed as nmoles of TBARS/mg of protein.

*(3) Quantification of Nonprotein Sulfhydryl Groups*. Quantification of nonprotein sulfhydryl (NPSH) was accomplished following Ellman [14]. Individual absorbance values were interpolated with glutathione standard curve and expressed as *µ*g of glutathione/g of protein.

*(4) Antioxidant Enzymatic Activities*. Catalase (CAT) activity was verified through the method described by Boveris and Chance [15], and results were expressed as nmoles/mg of protein. Superoxide dismutase (SOD) activity was evaluated as described by Boveris and Cadenas [16]. Results were expressed as units of SOD/mg of protein. Enzymatic activity of glutathione S-transferase (GST) was performed according to Habig et al. [17], and results were expressed in *μ*moles/min per mg of protein.

### 2.4. Protocol 2: Experimental Wound Healing Design

At the beginning of the experimental protocol (day 0), to induce the wound healing process along with inflammation and oxidative unbalance, a patch of the skin of 1 cm^2^ was removed from all animals. Each animal was anesthetized with the association of ketamine (75 mg/kg) and xylazine (10 mg/kg) administrated intraperitoneally [18]. After that, animals were immobilized at a surgical table, and procedure was conducted using surgical scissors and tweezers. Wounds were cut circularly in the middle of the upper back of each animal, about 20 mm from the base of the skull and normalized [19]. After this surgical procedure, total animals (*n* = 72) were separated into four groups:

  Control group: animals that did not receive any treatment, only saline by oral gavage and simulation of exposition to the magnetic field (*n* = 18)
  Group treated with cubiu: animals treated with the ingestion by oral gavage of 50 mg/kg of cubiu extract (*n* = 18)
  Group treated with magnetic field: animals treated by the exposition to the magnetic field (*n* = 18)
  Group treated with magnetic field + cubiu: animals treated with the ingestion by oral gavage of 50 mg/kg of cubiu extract and exposition to the magnetic field (*n* = 18)

**Figure 1:**
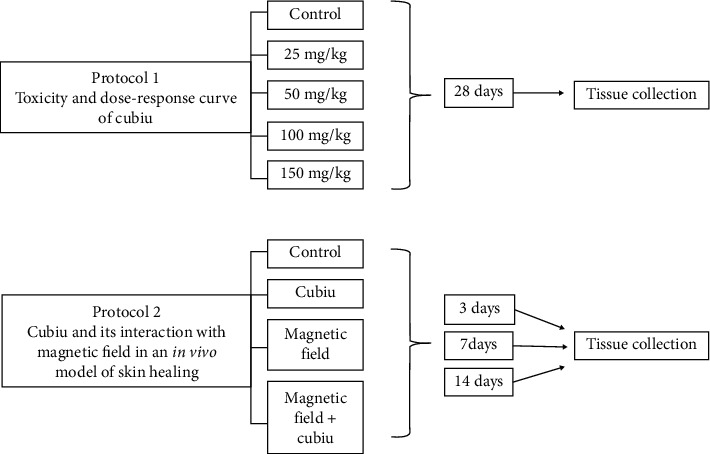
Schematic demonstration of the experimental procedures of protocols 1 and 2.

The 50 mg/kg cubiu extract dose used in the experiment was chosen according to results of toxicity studies. The pulsed magnetic field used in this experiment was a pulsed one, with a flux density of 2 mT, constant intervals between pulses (1, 5 s), frequency of 60 Hz, and 30 minutes of exposition per day, which was chosen based on the literature [20–22]. Treatments were performed once daily. On days 3, 7, and 14 after surgery, the animals (*n* = 6 per group) were euthanized to proceed with the collection of blood and skin samples [23, 24].  Figure 1 shows the experimental procedures of protocols 1 and 2.

#### 2.4.1. Inflammatory Cytokines

Skin and plasma determination of interleukin 1 (IL-1), interleukin 6 (IL-6), interleukin 10 (IL-10), and tumor necrosis factor-alpha (TNF-*α*) were evaluated using the ELISA kit (eBioscience, San Diego, EUA), according to the manufacturer's instructions. Hemocysteine levels were assayed using an IMMULITE analyzer (Diagnostic Products Corporation, Los Angeles, California). Results are expressed in pg/ml.

#### 2.4.2. Leukocyte Infiltration Marker

Skin samples were collected to evaluate the myeloperoxidase activity (MPO) to estimate inflammatory cell infiltration in the skin after injury. The activity of this enzyme was used as a marker of neutrophil infiltration [25, 26]. First, tissue samples were homogenized with a motor-driven homogenizer in 300 *μ*l of acetate buffer (8 mM, pH 5.4) containing 0.5% HTAB (45 s at 0°C). The homogenate was then centrifuged at 11000 ×g at 4°C for 20 min, and supernatants were stored at −4°C. To evaluate MPO activity, 10 *μ*l of supernatant was incubated with 200 *μ*l of acetate buffer (8 mM, pH 5.4) and 20 *μ*l of TMB (18.4 mM) at 37°C for 3 min. The reaction was stopped with 30 *μ*l of acetic acid in a cold bath, and the enzyme activity value was assessed colorimetrically at 630 nm using a microplate reader. Results were expressed as optical density (OD)/mg of protein.

#### 2.4.3. Determination of Oxidative Status

Fresh homogenized hepatic and dermal tissues (skin). TBARS and NPSH levels and SOD, CAT, and GST activities were determined as described in Sections 2.3.3

### 2.5. Statistical Analysis

For the first protocol, homogeneity of variances among different tested concentrations was verified with Levene's test and one-way ANOVA, followed by Tukey's post hoc test. For the second protocol, homogeneity of variances was evaluated in the same way, and a three-way ANOVA was performed followed by Tukey. The software used for this analysis was software Statistica® 7.0. The significance level (*α*) adopted was ≤0.05.

## 3. Results

### 3.1. Oral Toxicity Evaluation of Cubiu Extract

Initially, cubiu extract daily oral administration toxicity was evaluated through a dose-response curve for 28 days. No changes in GOT, GPT, GGT, glucose, triglycerides, total cholesterol, urea, and uric acid levels were observed between the groups that received different doses of cubiu extract in comparison to the control group (Table 1). Regarding creatinine levels, animals treated with cubiu extract at a dose of 100 mg/kg had their levels increased when compared to the animals treated with 25 mg/kg of cubiu extract.

The second objective of the experiments conducted in protocol 1 was to determine, among the studied doses, the one with the greatest beneficial potential in the face of oxidative status.  Table 2 provides the oxidative/antioxidant biomarkers in the liver, in which no change was observed in levels of TBARS, NPSH, and GST between the groups that received the different doses of cubiu extract compared to the control group. As for the enzymatic activity of SOD, an increase was observed in the groups treated with 25 mg/kg, 50 mg/kg, and 150 mg/kg of cubiu extract compared to the control group. In addition, the group treated with 100 mg/kg of cubiu extract presented reduced SOD activity compared to the groups treated with 25 mg/kg and 150 mg/kg of cubiu extract. The enzymatic activity of CAT was increased in the group treated with 25 mg/kg of cubiu extract compared to the control group and compared to the groups treated with 100 mg/kg and 150 mg/kg of cubiu extract.

For the renal biomarkers of oxidative stress (Table 2), it was evident that enzymatic activity of SOD was increased in the groups treated with 25 mg/kg, 50 mg/kg, and 100 mg/kg of cubiu extract compared to the control group. Furthermore, the enzymatic activity of CAT was increased in the group treated with 50 mg/kg of cubiu extract compared to the control group.

**Figure 2:**
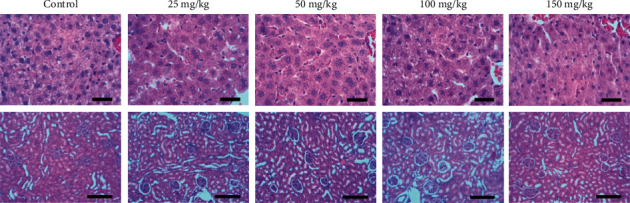
Photomicrography of the liver (L) and photomicrography of the kidney (K) of the Wistar rats in the control group, groups treated with 25 mg/kg of cubiu extract, 50 mg/kg of cubiu extract, 100 mg/kg of cubiu extract, and 150 mg/kg of cubiu extract. Bar = 20 micrometers in the liver's photomicrography and 100 micrometers in the kidney's photomicrography.

The morphological analysis showed no macroscopic or histological changes in hepatic and renal structures between the groups that received different doses of cubiu extract (25, 50, 100, and 150 mg/kg) compared to the control group (Figure 2).

Following the presented data, the dose of cubiu extract chosen for continuity of the other experimental protocols was 50 mg/kg.

### 3.2. Inflammatory Biomarkers in the Skin and Plasma

The results of inflammatory markers in the skin are given in Table 3 and in the plasma are given in Table 4. Levels of proinflammatory cytokines IL-1 and IL-6, in the skin and plasma, were decreased in all experimental groups on the 3^rd^ day in comparison to the control group of the respective time. Also, on 3^rd^ day, the TNF-*α* levels decreased in the skin and plasma in groups treated with magnetic field and with magnetic field + cubiu. On days 7 and 14, the group treated with magnetic field and the group treated with magnetic field + cubiu had decreased IL-1, IL-6, and TNF-*α* skin and plasma levels when compared to the control group. Additionally, on the 14^th^ day, the group treated with cubiu had decreased IL-6 plasma levels when compared to the control group.

Furthermore, among the treatments, it was observed that in the skin, on day 3, the group treated with magnetic field and cubiu combination significantly reduced IL-1, IL-6, and TNF-*α* levels when compared to the group treated with cubiu or magnetic field separately. On the 7^th^ day, the group treated with magnetic field + cubiu presented a reduction in IL-1 levels when compared to the group treated only with cubiu. On day 14, the group treated with the magnetic field and cubiu combination had reduced levels of IL-1 when compared to the groups treated with cubiu or magnetic field separately, and the group treated with magnetic field + cubiu presented a decrease in IL-6 and TNF-*α* levels when compared to the group treated only with cubiu. Additionally, IL-6 levels reduced in the group treated with magnetic field compared to the group treated with cubiu.

In plasma, among the treatments, it was observed that, on the 3^rd^ day, the group treated with the magnetic field and cubiu combination had decreased levels of IL-1, IL-6, and TNF-*α* when compared to the group treated with cubiu or with magnetic field separately. On days 7 and 14, the group treated with magnetic field + cubiu presented a decrease in IL-1 and Il-6 levels compared to the group treated only with cubiu. Also, on the 14^th^ day, TNF-*α* levels decreased in the group treated with magnetic field + cubiu compared to the group treated only with cubiu.

IL-10 anti-inflammatory cytokine levels in the skin and plasma were increased in the group treated with magnetic field and the group treated with magnetic field + cubiu on the 3^rd^ day in comparison to the control group of the respective time. On the 7^th^ day, the group treated with magnetic field + cubiu presented increased levels of IL-10 in comparison to the control group. On day 14, all treatments (cubiu, magnetic field, and magnetic field + cubiu) had higher levels of IL-10 when compared to the control group. Furthermore, among the treatments, it was observed that in the skin and plasma, on day 3, the group treated with magnetic field + cubiu had increased IL-10 levels in comparison to the group treated only with cubiu. On day 7, the group treated with the magnetic field and cubiu combination had increased IL-10 levels compared to the group treated with cubiu or with magnetic field separately.

Regarding the changes in IL-1, IL-6, and TNF-*α* proinflammatory cytokines skin and plasma levels over time, reductions were generally observed in all experimental groups. In relation to IL-10 anti-inflammatory cytokine skin and plasma levels over time, increases were generally observed in all experimental groups, corresponding to what was physiologically expected.

### 3.3. Leukocyte Infiltration Markers in the Skin

**Figure 3:**
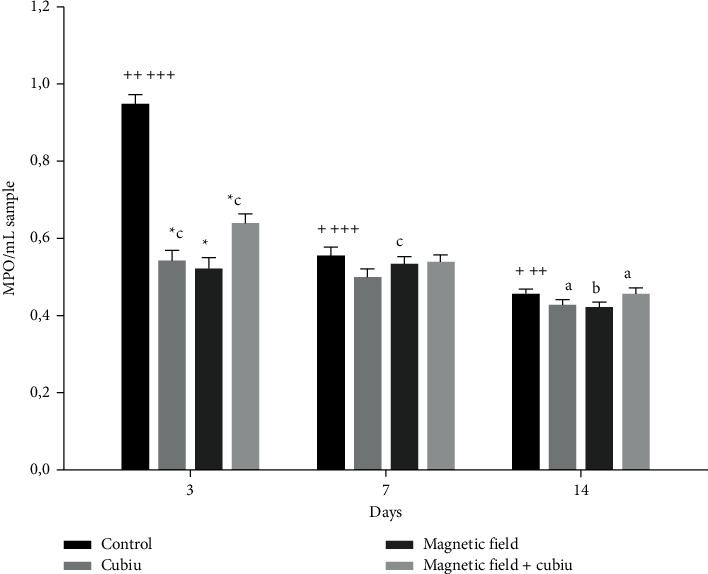
Graphical representation of myeloperoxidase activity. The results were expressed as optical density (OD)/mg of protein. ^*∗*^Significant differences from the control group at the same time. Lowercase letters indicate significant differences between times at the same treatment. The letters “a” corresponds to the group cubiu, “b” corresponds to the group MF, and “c” corresponds to the group MF + cubiu. To demonstrate a significant difference between the control groups of different times are used ^+^ for the control 3^rd^ day, ^++^ for the control 7^th^ day, and ^+++^ for the control 14^th^ day.

Results in myeloperoxidase activity are shown in Figure 3. On the 3^rd^ day, it was evident that all treatments (cubiu, magnetic field, and magnetic field + cubiu) decreased the myeloperoxidase enzymatic activity in comparison to the control group. In addition, over time, a decrease in enzymatic activity of myeloperoxidase was observed in the control group. The group treated with cubiu, and the group treated with magnetic field + cubiu presented a decrease in myeloperoxidase activity on the 14^th^ day compared to the 3^rd^ day. Also, the magnetic field group demonstrated a reduction in myeloperoxidase activity on the 14^th^ day compared to the 7^th^ day.

### 3.4. Oxidative Status in the Skin

Table 5 provides the results concerning oxidative status biomarkers in the skin. It was observed that the levels of TBARS were reduced in all treatments (cubiu, magnetic field, and magnetic field + cubiu) compared to the control group on the 3^rd^ day of treatment. On the 14^th^ day, the group treated with magnetic field had reduced TBARS levels when compared to the control group.

NPSH levels were increased in the group treated with magnetic field + cubiu on the 14^th^ day compared to the control group. In addition, changes over time were observed; the control group and the group treated with cubiu had reduced NPSH levels on day 7 when compared to the respective group on days 3 and 14. The group treated with magnetic field and the group treated with magnetic field + cubiu increased NPSH levels on the 14^th^ day compared to the 3^rd^ day and 7^th^ day.

As for SOD activity, it was observed that the group treated with magnetic field + cubiu had its activity increased on the 14^th^ day in relation to the control group. Also, changes were observed over time. In the control group, there was a reduction in SOD activity on day 7 compared to day 3, a reduction in the group treated with magnetic field on day 7 in comparison to days 3 and 14, and an increase in the group treated with magnetic field + cubiu on the 14^th^ day compared to the 7^th^ day.

CAT activity increased in the group treated with cubiu and the group treated with magnetic field + cubiu on the 14^th^ day compared to the 7^th^ day. Enzyme GST activity demonstrated an increase on the 14^th^ day compared to the 3^rd^ and 7^th^ days in all treated groups (cubiu, magnetic field, and magnetic field + cubiu), as well as in the control group.

## 4. Discussion

Cubiu is widely used for food and traditional medicine [1, 2]. Therefore, it is important to check the safety of cubiu toxicity. Experimental results regarding the toxicity of daily oral administration of cubiu extract evaluated through a dose-response curve for 28 days demonstrated the safety of cubiu extract. Biochemical parameters indicated that the extract did not present toxicity at any dose evaluated in this study. Furthermore, macroscopic and microscopic (histology) assessments of the liver and kidney, considered indispensable for toxicological analysis, evidenced that tested doses preserved the normal structure of these organs. In agreement with our results, Hernandes et al. [27] reported safe use of cubiu due to the absence of genotoxic and cytotoxic effects.

The dose of 50 mg/kg was chosen for the second protocol in this study; it did not present toxicity, and it revealed benefits to oxidative status, increasing the enzymatic activity of SOD in the liver and increasing enzymatic activity of SOD and CAT in the kidney.

After choosing cubiu extract dose, we followed a standardized wound healing model in the second protocol, also operating as an induction model for inflammatory and oxidative unbalance in the second protocol. This choice finds legitimacy in literature [19]. Magnetic fields have testified wound healing effects [28, 29], and *Solanum sessiliflorum* has its popular use as a wound healing, but there is no scientific confirmation [30].

In a wound healing model, it is important to conduct evaluations at the 3^rd^, 7^th^, and 14^th^ day of treatment after injury induction. This strategy is very important to estimate the effect of treatments at each of the different phases of the wound healing process, divided into three distinguished but overlapping stages [31]. The first phase is the inflammatory phase, marked by the presence of inflammatory cells and mediators, occurring 0–3 days after initial injury. The next phase is known as proliferation, characterized by the presence of fibroblasts and synthesis of the extracellular matrix. This phase takes place from 3^rd^ to 7^th^ day. The last phase is tissue remodeling, marked by the reorganization of the extracellular matrix and maturation of collagen fibers. Didactically, remodeling happens from 7^th^ to 14^th^ days, but it can persist for months [31, 32].

It is well known that wound healing treatments operate mainly at the first stage of healing, the inflammatory phase, contributing to repair due to anti-inflammatory and antioxidant properties of drugs [29]. Eventhough magnetic fields are described in the literature as a treatment for skin wound healing, their contribution to systemic inflammatory and oxidant profile is still controversial. Depending on the application parameters such as time and intensity, magnetic fields can be prooxidant or antioxidant, as well as be anti-inflammatory or promote inflammation in other tissues [33–36]. Considering that, it has been suggested that the systemic use of antioxidant substances along with magnetic fields should be investigated [6, 7]. Therefore, we analyzed the action of these treatments not only on the skin but also on plasma to evaluate the response towards the systemic inflammatory profile.

Observing the results from all treatments proposed on the inflammatory profile, it was evidenced that all treatments, isolated (cubiu or magnetic field) and combined (magnetic field + cubiu), demonstrated anti-inflammatory activity, and the anti-inflammatory action is increased with the combination of cubiu and magnetic field. Treatments were able to reduce the levels of proinflammatory cytokines and increase the levels of anti-inflammatory cytokine (IL-10), both on the skin and plasma. Inflammatory cytokines are mediators present during the wound healing process. They can act by increasing the spread of the inflammatory process, as is the case of IL-1, IL-6, and TNF-*α*, and they can also act by reducing and controlling the inflammatory process, as is the case of IL-10 [32].

Thus, an increase in the levels of proinflammatory cytokines during healing ends up delaying this healing process, in addition to causing damage at the systemic level, as is the case of serious injuries or infections, in which there may be hemodynamic and metabolic changes, organ damage, and even compromised life. In this context, anti-inflammatory cytokines act by containing and controlling the inflammatory process [37].

The action of the magnetic field in the reduction of proinflammatory cytokines is justified by its action mechanism that acts on the modulation of adenosine and its receptors A_2A_ and A_3_ARs, increasing the expression of these, which are capable of suppressing high levels of proinflammatory cytokines [38]. Also, the anti-inflammatory activity of cubiu can be based on the bioactive compounds present in its composition. The cubiu extract used in this study had its phytochemical characterization previously published in [9], evidencing the presence of phenolic compounds in its composition.

In agreement, Rodrigues et al. [39] demonstrated through HPLC-MS that cubiu extract constituted mainly of phenolic compounds, such as chlorogenic acid, specially 5-caffeoylquinic acid. Also, other species of *Solanum*, such as *Solanum lycocarpum*, has phenolic compounds, including caffeoylquinic acids in its composition, to which antioxidant and anti-inflammatory capacities exerted by Solanaceae were attributed [40]. The anti-inflammatory activity exerted by phenolic compounds has as its mechanism of action the ability to inhibit enzymes that are related to inflammation, such as prostaglandin synthetase, lipoxygenase, and cyclooxygenase [41]. In addition, chlorogenic acid interferes with the leukocyte response to chemokines, preventing their interaction with the adhesion molecules involved in cell migration, thus inhibiting cell adhesion and controlling and reducing the inflammatory process [42].

In relation to cell migration, this research also explored the effect of treatment towards the enzymatic activity of myeloperoxidase. This enzyme is known as a biomarker for neutrophil infiltrations [43]. Our results demonstrated that all treatments reduced the activity of myeloperoxidase on the 3^rd^ day, which corresponds to the inflammatory phase of the wound healing process. It indicates that treatments promoted a reduction in neutrophilic infiltration, reinforcing anti-inflammatory properties of magnetic field and cubiu. This corroborates previous results of the present study regarding the determination of cytokines, which demonstrated reduced levels of proinflammatory cytokines and increased levels of anti-inflammatory cytokines for treated groups.

During the wound healing process, intimately related to inflammation, reactive oxygen species (ROS) and reactive nitrogen species (RNS) are released, which play a defensive role, especially against potential microbial invasion, and act on cell signaling in the healing process. However, when the excessive formation of reactive species overcomes the body's antioxidant capacity, a condition known as oxidative stress is generated. Oxidative stress can lead to damage on the cell membrane, lipids, proteins, and DNA and may culminate in cellular death [44–46]. Also, oxidative stress acts on the propagation of the inflammatory response because the production of inflammatory enzymes such as cyclooxygenase (COX) and lipoxygenase is stimulated from lipid peroxidation and induces leukocytes to release proinflammatory cytokines [47]. Thus, it is well described how oxidative stress is closely related to the pathogenesis of nonhealing wounds. Therefore, excess in reactive species must be avoided because it slows down the healing process [48, 49].

Results demonstrate that treatments proved to maintain oxidative balance with safety, manifesting a clear antioxidant potential. That becomes evident with the reduction of TBARS (biomarker for lipoperoxidation) in the skin, promoted by all treatments on day 3. The decrease of these substances in inflammatory phase is very positive, considering the great influence of reactive species in this period [49]. Thus, all treatments showed protection against lipoperoxidation.

On day 14, treatment with magnetic field reduced TBARS and treatment with magnetic field + cubiu increased NPSH and SOD levels in the skin. Therefore, treatments positively improved the antioxidant defense system. Differences throughout time are expected as part of the natural wound healing process, both for oxidative and inflammatory profile, as the quantification of analyses oscillates towards balance. Again, the combination of magnetic field + cubiu revealed positive results. It is known that magnetic fields promote exchanges with the biomagnetic potential of the organism, thus being able to activate enzymatic reactions [50, 51]. The action of magnetic fields in oxidative balance can occur through the cellular signaling mechanism, such as the extracellular signal-regulated kinase pathways [52]. Also, the antioxidant activity of cubiu can be attributed to the presence of phenolic compounds in its composition, which are known for their antioxidant and chelant properties [53].

Thereby, the results from this study allow us to affirm that cubiu can be used safely, not demonstrating toxicity at the researched doses and during the period of administration. We evidenced that isolated (only magnetic field or cubiu) or combined treatments (magnetic field + cubiu) manifested anti-inflammatory and antioxidant properties, improving all stages of the wound healing process, especially the inflammatory phase. The association of the treatments has better results than when used alone.

## 5. Conclusion

This research revealed that cubiu extract demonstrated safety and absence of toxicity at the administrated doses during the time of treatment adopted in our protocol. It also enlightened that the proposed treatments provided decreased levels of proinflammatory cytokines, increased levels of anti-inflammatory cytokines, and reduced activity of myeloperoxidase. Concerning stress biomarkers, treatments reduced TBARS levels and increased enzymatic activity of SOD and NPSH levels. Therefore, cubiu promotes antioxidant and anti-inflammatory actions in skin wound healing and increases the results of conventional treatment for skin healing (magnetic field) when used in association.

## Figures and Tables

**Table 1:** Biochemical parameters of rats after 28-day treatment with cubiu extract.

	Control	25 mg/kg	50 mg/kg	100 mg/kg	150 mg/kg
GOT (UI/l)	151.02 ± 36.00	179.83 ± 16.72	159.75 ± 31.11	151.90 ± 15.14	172.27 ± 11.71
GPT (UI/l)	132.98 ± 9.04	108.83 ± 12.30	147.82 ± 7.63	117.26 ± 17.12	123.96 ± 16.06
GAMA GT (U/l)	28.73 ± 3.19	25.14 ± 1.56	34.83 ± 5.97	34.47 ± 3.29	24.77 ± 5.53
Glucose (g/dl)	100.54 ± 12.64	140.35 ± 17.12	121.79 ± 8.91	141.43 ± 9.01	105.22 ± 20.75
Triglycerides (mg/dl)	125.92 ± 14.37	108.33 ± 28.09	110.18 ± 21.35	108.33 ± 23.29	78.70 ± 2.44
Total cholesterol (mg/dl)	87.61 ± 21.96	88.57 ± 24.41	58.09 ± 11.23	116.18 ± 9.94	73.33 ± 20.88
Urea (mg/dl)	52.06 ± 3.07	44.95 ± 3.69	43.24 ± 4.93	46.37 ± 2.05	44.38 ± 3.84
Creatinine (mg/dl)	0.57 ± 0.004	0.53 ± 0.06^D^	0.55 ± 0.02	0.73 ± 0.05^B^	0.60 ± 0.01
Uric acid (mg/dl)	1.43 ± 0.31	0.79 ± 0.13	1.17 ± 0.40	1.46 ± 0.40	0.95 ± 0.24

Values are represented as the mean ± standard error. Different capital letters indicate significant differences between treatments (*p* < 0.05). The capital letters “B” corresponds to the group 50 mg/kg and letter “D” corresponds to the group 150 mg/kg. GOT, glutamic oxaloacetic transaminase; GPT, glutamic pyruvic transaminase; GAMA GT, gamma-glutamyl transpeptidase.

**Table 2:** Biomarkers of oxidative status in the liver and kidney after 28-day treatment with cubiu extract.

	Liver					Kidney				
	Control	25	50	100	150	Control	25	50	100	150
TBARS	0.91 ± 0.24	0.83 ± 0.21	0.81 ± 0.13	0.80 ± 0.12	1.20 ± 0.13	0.72 ± 0.04	1.71 ± 0.51	1.40 ± 0.37	1.30 ± 0.37	0.74 ± 0.08
NPSH	4.06 ± 0.14	3.99 ± 0.71	3.32 ± 0.44	2.87 ± 0.16	4.10 ± 0.59	1.81 ± 0.20	2.07 ± 0.21	2.06 ± 0.26	1.83 ± 0.26	1.61 ± 0.19
SOD	4.58 ± 0.41	9.29 ± 0.31^*∗*^^C^	8.03 ± 0.63^*∗*^	6.64 ± 0.39^AD^	9.78 ± 1.06^*∗*^^C^	5.34 ± 0.21	7.69 ± 0.53^*∗*^	7.17 ± 0.14^*∗*^	8.02 ± 0.77^*∗*^	6.47 ± 0.41
CAT	3.14 ± 0.33	5.56 ± 0.81^*∗*^^CD^	3.61 ± 0.51	3.47 ± 0.45^A^	2.96 ± 0.22^A^	1.14 ± 0.07	1.27 ± 0.20	1.78 ± 0.16^*∗*^	1.67 ± 0.21	1.31 ± 0.10
GST	6.94 ± 1.12	11.50 ± 1.23	9.06 ± 1.30	6.67 ± 1.00	8.65 ± 2.93	1.16 ± 0.09	1.25 ± 0.22	1.08 ± 0.12	1.34 ± 0.24	1.17 ± 0.04

Values are represented as the mean ± standard error. ^*∗*^Significant differences from the control group (*p* < 0.05). Different capital letters indicate significant differences between treatments (*p* < 0.05). The capital letter “A” corresponds to the group 25 mg/kg, “B” corresponds to the group 50 mg/kg, “C” corresponds to the group 100 mg/kg, and letter “D” corresponds to the group 150 mg/kg. TBARS, thiobarbituric acid reactive substances (*µ*mol/mg protein); SPSH, nonprotein thiols (nmol/mg protein); SOD, superoxide dismutase (SOD units/mg protein); CAT, catalase (*ρ*mol/mg protein); GST, glutathione S-transferase (*ρ*mol/min/mg protein).

**Table 3:** Inflammatory biomarkers in the skin.

Time	Treatments	IL-1	IL-6	TNF-*α*	IL-10
3 day	Control	90.67 ± 0.84^++ +++^	99.17 ± 1.14^++ +++^	105.33 ± 1.69^++ +++^	20.25 ± 1.22^++ +++^
	Cubiu	84.00 ± 0.36^*∗*^^bcC^	89.67 ± 0.67^*∗*^^bcC^	101.67 ± 1.26^bcC^	26.70 ± 1.65^cC^
	Magnetic field	82.83 ± 0.60^*∗*^^bcC^	89.00 ± 1.39^*∗*^^bcC^	95.67 ± 1.28^*∗*^^bcC^	30.28 ± 2.05^*∗*^^c^
	Magnetic field + cubiu	71.33 ± 0.88^*∗*^^ABc^	75.83 ± 1.40^*∗*^^ABc^	82.83 ± 1.58^*∗*^^ABc^	36.01 ± 1.64^*∗*^^Ac^

7 day	Control	76.40 ± 1.50^+ +++^	81.33 ± 0.84^+ +++^	88.17 ± 0.48^+ +++^	33.15 ± 0.90^+ +++^
	Cubiu	74.33 ± 1.11^acC^	79.33 ± 1.65^ac^	85.00 ± 1.15^ac^	31.89 ± 0.82^cC^
	Magnetic field	70.50 ± 0.84^*∗*^^ac^	73.50 ± 1.34^*∗*^^ac^	79.67 ± 0.84^*∗*^^ac^	31.35 ± 1.05^cC^
	Magnetic field + cubiu	68.20 ± 1.77^*∗*^^Ac^	73.17 ± 1.60^*∗*^^c^	80.67 ± 1.58^*∗*^^c^	40.49 ± 1.77^*∗*^^ABc^

14 day	Control	58.80 ± 1.24^+ ++^	70.50 ± 0.72^+ ++^	77.00 ± 1.24^+ ++^	47.12 ± 1.37^+ ++^
	Cubiu	55.20 ± 1.24^abC^	64.75 ± 2.50^abBC^	72.17 ± 1.74^abC^	55.54 ± 1.26^*∗*^^ab^
	Magnetic field	53.00 ± 0.51^*∗*^^abC^	57.20 ± 1.46^*∗*^^aAb^	67.17 ± 1.64^*∗*^^ab^	60.20 ± 0.73^*∗*^^ab^
	Magnetic field + cubiu	46.50 ± 1.19^*∗*^^aAbB^	53.17 ± 1.01^*∗*^^aAb^	62.83 ± 1.64^*∗*^^aAb^	60.92 ± 0.95^*∗*^^ab^

Values are represented as the mean ± standard error. ^*∗*^Significant differences from the control group at the same time (*p* < 0.05). Different capital letters indicate significant differences between treatments at the same time (*p* < 0.05). Different lowercase letters indicate significant differences between times at the same treatment (*p* < 0.05). The capital letters “A” corresponds to the group cubiu, “B” corresponds to the group MF, and “C” corresponds to the group MF + cubiu. The lowercase letters “a” corresponds to the time 3^rd^ day of the respective group, “b” corresponds to the time 7^th^ day of the respective group, and “c” corresponds to the time 14^th^ day of the respective group. To demonstrate the significant difference between the control groups of different times are used: ^+^control 3^rd^ day, ^++^control 7^th^ day, and ^+++^control 14^th^ day.

**Table 4:** Inflammatory biomarkers in plasma.

Time	Treatments	IL-1	IL-6	TNF-*α*	IL-10
3 day	Control	150.33 ± 1.43^++ +++^	165.50 ± 2.08^++ +++^	175.67 ± 2.88^++ +++^	18.83 ± 1.14^++ +++^
	Cubiu	139.33 ± 0.71^*∗*^^bcC^	149.33 ± 1.23^*∗*^^bcC^	170.00 ± 2.07^bcC^	24.83 ± 1.54^cC^
	Magnetic field	137.17 ± 1.01^*∗*^^bcC^	148.00 ± 1.98^*∗*^^bcC^	159.50 ± 2.39^*∗*^^bcC^	28.17 ± 1.90^*∗*^^c^
	Magnetic field + cubiu	117.67 ± 1.89^*∗*^^ABc^	128.60 ± 1.69^*∗*^^ABc^	139.17 ± 2.50^*∗*^^ABc^	33.50 ± 1.52^*∗*^^Ac^

7 day	Control	127.50 ± 2.17^+ +++^	135.83 ± 1.40^+ +++^	147.50 ± 0.76^+ +++^	30.83 ± 0.83^+ +++^
	Cubiu	123.00 ± 1.57^acC^	132.00 ± 2.76^acC^	141.67 ± 1.54^ac^	29.67 ± 0.76^cC^
	Magnetic field	115.33 ± 1.80^*∗*^^ac^	124.60 ± 1.72^*∗*^^ac^	133.50 ± 1.73^*∗*^^ac^	29.17 ± 0.98^cC^
	Magnetic field + cubiu	112.50 ± 2.95^*∗*^^Ac^	121.67 ± 2.69^*∗*^^Ac^	134.67 ± 2.64^*∗*^^c^	37.67 ± 1.65^*∗*^^ABc^

14 day	Control	99.50 ± 2.26^+ ++^	117.83 ± 1.11^+ ++^	128.17 ± 1.94^+ ++^	43.83 ± 1.28^+ ++^
	Cubiu	91.50 ± 1.84^abC^	106.00 ± 3.99^*∗*^^abC^	120.50 ± 2.88^abC^	51.67 ± 1.17^*∗*^^ab^
	Magnetic field	87.83 ± 1.01^*∗*^^ab^	95.40 ± 2.52^*∗*^^ab^	112.00 ± 2.65^*∗*^^ab^	56.00 ± 0.68^*∗*^^ab^
	Magnetic field + cubiu	80.17 ± 2.85^*∗*^^aAb^	88.67 ± 1.73^*∗*^^aAb^	105.00 ± 2.65^*∗*^^aAb^	56.67 ± 0.88^*∗*^^ab^

Values are represented as the mean ± standard error. ^*∗*^Significant differences from the control group at the same time (*p* < 0.05). Different capital letters indicate significant differences between treatments at the same time (*p* < 0.05). Different lowercase letters indicate significant differences between times at the same treatment (*p* < 0.05). The capital letters “A” corresponds to the group cubiu, “B” corresponds to the group MF, and “C” corresponds to the group MF + cubiu. The lowercase letters “a” corresponds to the time 3rd day of the respective group, “b” corresponds to the time 7th day of the respective group, and “c” corresponds to the time 14th day of the respective group. To demonstrate a significant difference between the control groups of different times are used: ^+^control 3rd day, ^++^control 7th day, and ^+++^control 14th day.

**Table 5:** Biomarkers of oxidative stress in the skin.

Time	Treatments	TBARS	NPSH	SOD	CAT	GST
3 day	Control	2.80 ± 0.16	16.7 ± 1.55^++^	4.93 ± 0.25^++^	4.48 ± 0.46	2.19 ± 0.23^+++^
	Cubiu	1.78 ± 0.07^*∗*^	16.1 ± 1.09^b^	4.19 ± 0.29	4.56 ± 0.62	1.82 ± 0.44^c^
	Magnetic field	1.73 ± 0.16^*∗*^	14.7 ± 0.94^c^	4.42 ± 0.37^b^	4.43 ± 0.53	0.85 ± 0.06^c^
	Magnetic field + cubiu	1.81 ± 0.12^*∗*^	15.8 ± 1.23^bc^	3.93 ± 0.40	3.34 ± 0.37	1.17 ± 0.38^c^

7 day	Control	2.17 ± 0.11	10.9 ± 0.44^++++^	2.91 ± 0.10^+^	2.84 ± 0.21	1.79 ± 0.15^+++^
	Cubiu	2.02 ± 0.12	10.7 ± 0.79^ac^	3.40 ± 0.25	2.90 ± 0.24^c^	2.35 ± 0.52^c^
	Magnetic field	1.52 ± 0.12	10.4 ± 0.32^c^	2.86 ± 0.23^ac^	3.13 ± 0.40	1.57 ± 0.25^c^
	Magnetic field + cubiu	1.93 ± 0.15	10.7 ± 0.70^ac^	2.76 ± 0.24^c^	3.16 ± 0.30^c^	1.55 ± 0.07^c^

14 day	Control	2.81 ± 0.07	17.2 ± 1.10^++^	3.77 ± 0.24	3.95 ± 0.32	5.25 ± 0.44^+ ++^
	Cubiu	2.36 ± 0.08	18.1 ± 1.28^b^	3.92 ± 0.14	4.77 ± 0.31^b^	4.70 ± 0.35^ab^
	Magnetic field	1.52 ± 0.12^*∗*^	21.3 ± 1.70^ab^	4.88 ± 0.39^b^	3.93 ± 0.42	4.14 ± 0.19^ab^
	Magnetic field + cubiu	2.39 ± 0.17	23.0 ± 1.24^*∗*^^ab^	5.43 ± 0.42^*∗*^^b^	5.00 ± 0.43^b^	5.31 ± 0.26^ab^

Values are represented as the mean ± standard error. ^*∗*^Significant differences from the control group at the same time (*p* < 0.05). Different capital letters indicate significant differences between treatments at the same time (*p* < 0.05). Different lowercase letters indicate significant differences between times at the same treatment (*p* < 0.05). The capital letters “A” corresponds to the group cubiu, “B” corresponds to the group MR, and “C” corresponds to the group MR + cubiu. The lowercase letters “a” corresponds to the time 3^rd^ day of the respective group, “b” corresponds to the time 7^th^ day of the respective group, and “c” corresponds to the time 14^th^ day of the respective group. To demonstrate a significant difference between the control groups of different times are used: ^+^control 3^rd^ day, ^++^control 7^th^ day, and ^+++^control 14^th^ day. TBARS, thiobarbituric acid reactive substances (*µ*mol/mg protein); SPSH, nonprotein thiols (nmol/mg protein); SOD, superoxide dismutase (SOD units/mg protein); CAT, catalase (*ρ*mol/mg protein); GST, glutathione S-transferase (*ρ*mol/min/mg protein).

## Data Availability

The data generated or analyzed during this study are included within this article.

## Additional Points

After the conclusions obtained in this study, we now intend to develop a nanostructured topical application of the cubiu and thus test its performance in skin wound healing.
